# Fluorescence Excitation and Dispersed Fluorescence Spectra of the First Electronic Excited (S_1_) State of *peri*-Hexabenzocoronene (C_42_H_18_) Isolated in Solid *para*-Hydrogen

**DOI:** 10.1021/acs.jpca.4c02320

**Published:** 2024-06-12

**Authors:** Isabelle Weber, Yuan-Pern Lee

**Affiliations:** † Department of Applied Chemistry and Institute of Molecular Science, National Yang Ming Chiao Tung University, Hsinchu 300093, Taiwan; ‡ Center for Emergent Functional Matter Science, National Yang Ming Chiao Tung University, Hsinchu 300093, Taiwan

## Abstract

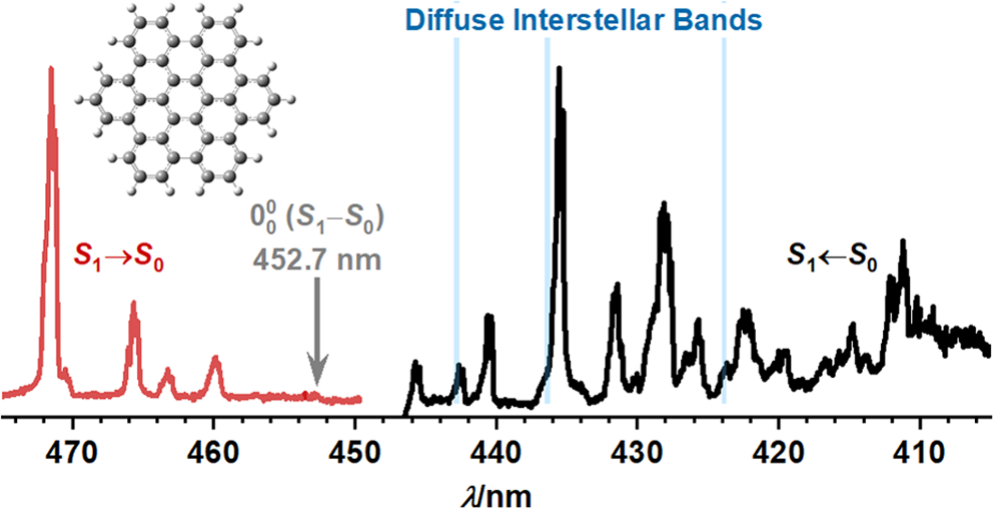

Large polycyclic aromatic hydrocarbons (PAH) and their cationic, hydrogenated, and protonated derivatives have long been considered as promising candidates for the carriers of the diffuse interstellar bands. *peri*-Hexabenzocoronene (*peri*-HBC, C_42_H_18_) is a large, compact PAH, and, to the best of our knowledge, the largest centrosymmetric all-benzenoid PAH for which electronic spectroscopy data has been published. In this work, we present the dispersed fluorescence and fluorescence excitation spectra of the first electronic excited (S_1_) state of *peri*-HBC isolated in solid *para*-H_2_ and provide the first detailed vibronic analysis of observed features. The observed spectra agree with the emission and absorption spectra simulated according to optimized geometries and scaled harmonic vibrational frequencies calculated at the density functional theory (DFT) level using a Franck–Condon Herzberg–Teller approach; the spectral bands are associated solely with vibrational normal modes of approximate e_2g_ symmetry and their combinations with vibrational modes of approximately a_1g_ symmetry. We clearly observed the position of the S_1_–S_0_ electronic transition origin of *peri*-HBC at 22,088 cm^–1^ (452.7 nm), which was unreported previously. The matrix shift of ∼110 cm^–1^ to the red relative to the gas-phase value was estimated by comparison of two reported gas-phase bands with our work. Because of the significant deviation from the reported wavelengths of DIB, the weakness of the S_1_–S_0_ electronic transitions, and the lack of reported DIB at <400 nm where the intense S_4_ ← S_0_ band of *peri*-HBC is located, *peri*-HBC is unlikely to contribute to DIB.

## 1 Introduction

Since the discovery of the first diffuse interstellar bands (DIB) by Heger in 1922,^1^ well over 500 DIBs have been confirmed.^2,3^ Nonetheless, despite their great numbers, the carrier molecules remain a mystery. So far, only the buckminsterfullerene cation C_60_^+^ has been successfully identified as the carrier of five DIB at 957.74, 936.57, 934.85, 942.84, and 963.26 nm, based on laboratory gaseous spectra recorded with a cryogenic ion trap.^4,5^ Large polycyclic aromatic hydrocarbons (PAH), their cations, and their protonated and hydrogenated derivatives are considered as particularly promising candidates for the DIB carriers;^6−8^ laboratory reference spectra of these species suitable for a comparison to astronomical observations, however, are rarely available.

**Figure 1:**
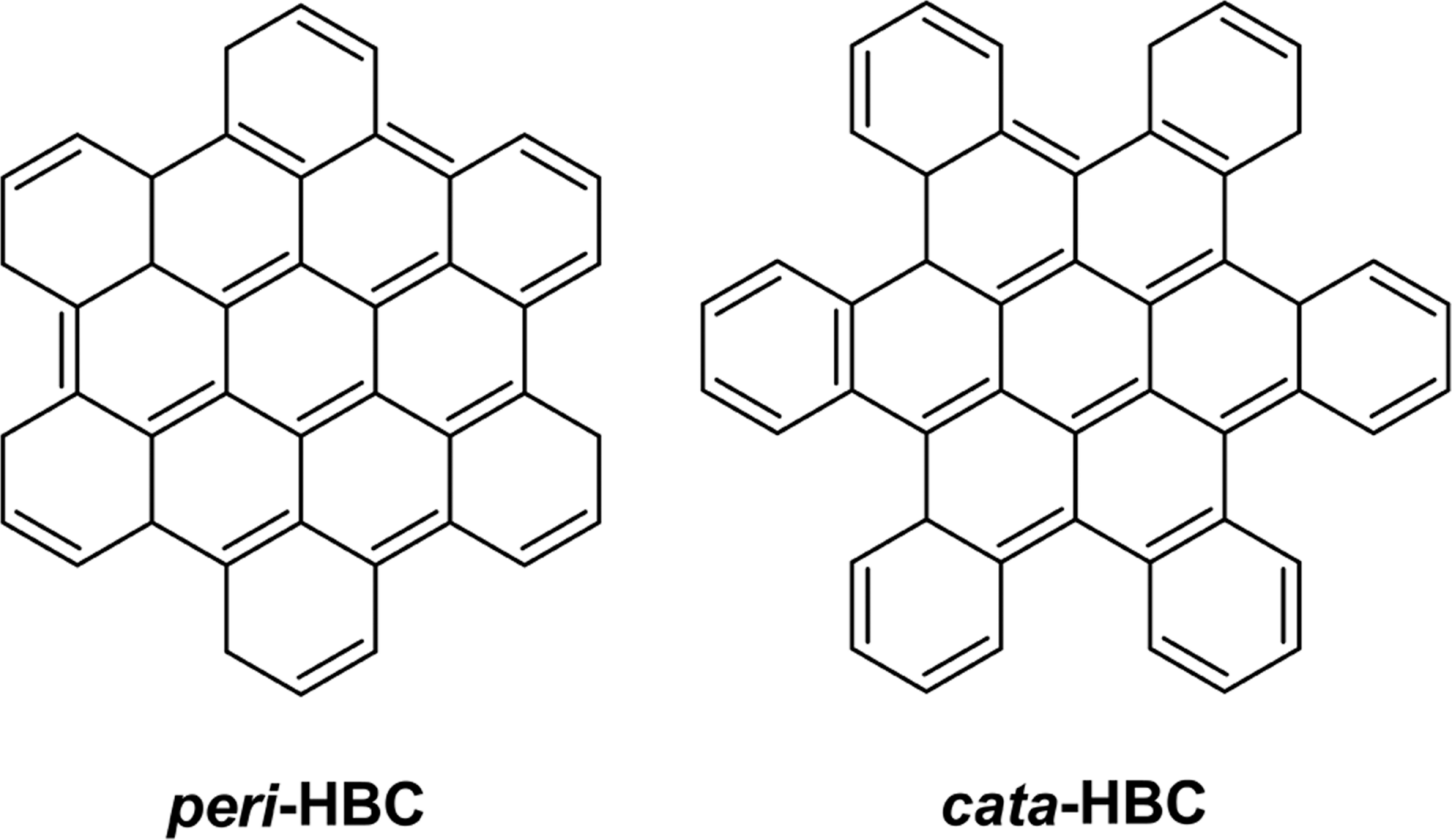
Molecular structures of the two hexabenzocoronene (HBC, C_42_H_18_) isomers *peri*-HBC and *cata*-HBC.

Hexabenzocoronene (HBC, C_42_H_18_) has two isomers *peri*-HBC and *cata*-HBC with *D*_6*h*_ symmetry, as shown in Figure 1. *peri*-HBC was first synthesized by Clar et al.^9^ in 1959. Due to its unique electronic properties, *peri*-HBC has been discussed *inter alia* as a material for semiconductors, solar cells, and Li-ion battery electrodes.^10−13^ With its compact structure, *peri*-HBC is regarded to be rather stable under the harsh conditions of the interstellar medium. According to the simulations by Le Page et al.,^14^ photolytic C_2_H_2_- and H-loss rates of PAH decrease with increasing carbon number (*N*_C_), and, a typical interstellar ultraviolet (UV) radiation field is too small to destroy PAH with *N*_C_ > 42. *peri*-HBC has, hence, been considered as a potential carrier for the DIB and the unidentified infrared (UIR) bands. Therefore, electronic absorption, fluorescence, phosphorescence, as well as infrared multiphoton dissociation (IRMPD) spectra of *peri*-HBC, *peri*-HBC cation, and protonated *peri*-HBC in a variety of environments have been reported.^9,15−24^

Bräuchle^18^ discussed the extent of *pseudo*-Jahn–Teller distortion in the triplet *T*_1_ state of *peri*- and *cata*-HBC, and, for this purpose, recorded the luminescence emission and optical microwave double resonance (OMDR) spectra of *peri*-HBC in epoxide at 1.3 K; he emphasized the similarity of *peri*-HBC to coronene and benzene, pointing out the identical symmetries of their first excited singlet and triplet states, and the, in consequence, similar phosphorescence spectra consisting of predominantly e_2g_ symmetric C–C stretching and bending modes. The similarity of coronene and *peri*-HBC was further elucidated by Fetzer and Zander,^19^ who reported fluorescence and phosphorescence emission spectra in perhydrocoronene at *T* = 77–353 K. The first assessment of *peri*-HBC as potential DIB carrier was reported by Hendel et al.^23^ in 1986. These authors presented a novel synthesis for *peri*-HBC and provided its fluorescence, phosphorescence, and UV absorption spectra in dioxane at 77 K, the IR spectrum of solid *peri*-HBC, and a photoelectron spectrum at 450 °C (gas-phase); no definite conclusions regarding the possibility of *peri*-HBC to be the carrier of DIB could be drawn.

Electronic absorption spectra of *peri*-HBC in solid Ar at 6 K and in solid Ne (5.8 K) and Ar (12 K) were reported by Steglich et al.^20^ and Rouillé et al.,^21^ respectively. As part of their study of the UV absorption spectra of PAH mixtures to analyze the UV bump near 217 nm commonly observed in astronomical observations, Steglich et al.^20^*inter alia* recorded the UV absorption spectra of coronene and *peri*-HBC isolated in solid Ar and as films at room temperature, and compared these to simulated spectra calculated using the ZINDO (Zerner’s model of intermediate neglect of differential overlap) method; no detailed analysis of the absorption spectra was provided. Rouille et al.^21^ assigned the absorption bands reported by Clar^9^ in 1959 to individual electronic transitions based on a comparison to their spectra of *peri*-HBC recorded by Ar and Ne matrix isolation techniques and quantum-chemical calculations with TD-DFT (B3LYP/6-311G) and ZINDO. They pointed out that, consistent with earlier theoretical studies,^25,26^ only DFT calculations at the B3LYP/6-311G level converge to a minimum structure with the *D*_6*h*_ symmetry. Peak positions of observed individual bands were specified; however, no assignments to distinct vibrational normal modes of *peri*-HBC were provided. To the best of our knowledge, the only electronic spectrum of *peri*-HBC in the gas phase was reported by Kokkin et al.^22^ The resonant 2-color-2-photon ionization (2C2PI) spectrum of jet-cooled *peri*-HBC with its most intense peak at 426.41 nm was used to estimate an upper limit for the abundancy of *peri*-HBC in the interstellar medium, 4 × 10^12^ cm^–2^. No in-depth analysis including the transition origin of the spectrum was attempted and no matching DIBs could be identified.

A major challenge in the identification of DIB carriers is the lack of laboratory reference spectra of large PAH and their derivatives suitable for comparison to astronomical observations. Because of the low vapor pressure of PAH, obtaining low-temperature gas-phase spectra of large PAH and their derivatives is challenging. The matrix isolation method, which requires significantly lower concentrations, is more feasible to obtain suitable spectra, but might suffer from severe and arbitrary shifts in line positions due to interactions of PAH with the matrix host. *para*-Hydrogen (*para*-H_2_) has been frequently employed as a matrix host to record the IR absorption spectra of PAH and their protonated and hydrogenated derivatives; see e.g., refs (27,28) and references cited therein. Consistent with the “softness” of the quantum solid *para*-H_2_, these spectra show only small matrix shifts for vibrational modes. However, the data for electronic transitions are rare. Over the past years, we have attempted to characterize *para*-H_2_ as a matrix host for electronic spectroscopy by recording dispersed fluorescence and fluorescence excitation spectra of several PAH for which gas-phase spectra at low temperatures are available in the literature.^29,30^ Our preliminary results indicate consistent redshifts below 100 cm^–1^ relative to the gas phase for neutral PAH from pyrene (C_16_H_10_) up to ovalene (C_32_H_14_).

In this contribution, we present the dispersed fluorescence and fluorescence excitation spectra of the first electronic excited (S_1_) state of *peri*-HBC isolated in solid *para*-H_2_, thereby extending our series of studied PAH to a PAH containing 42 C atoms, the largest neutral centrosymmetric all-benzenoid PAH for which low-temperature gas-phase spectra are available in the literature. We compare our experimental data to simulated spectra obtained by a Franck–Condon Herzberg–Teller approach on the basis of calculations with the (TD-)B3PW91/6-311++G(2d,2p) and (TD-)wB97xD/6-311+G(d,p) methods, and derive the first detailed vibronic assignments associated with the S_1_–S_0_ transition of *peri*-HBC. A comparison to the spectrum reported by Kokkin et al.^22^ indicates a matrix red shift of ∼110 cm^–1^ induced by the *para*-H_2_ environment, consistent with our earlier works.^29,30^ The use of *para*-H_2_ in the quest for the carriers of the DIB and the relevance of *peri*-HBC as a potential DIB carrier are briefly discussed.

## 2 Methods

Our *para*-H_2_ matrix isolation/laser-induced fluorescence setup has been described in detail elsewhere;^29−31^ therefore, only a brief summary is given here. The sample substrate, a nickel-plated copper flat mounted to the second stage of a closed-cycle helium refrigerator (Sumitomo F-50 compressor), is cooled to ∼3 K and serves as a matrix substrate and a reflective surface for spectroscopy. To characterize the sample, infrared spectra were recorded using a Fourier transform infrared spectrometer (FTIR, Bruker IFS66v) equipped with a KBr beam splitter and a Hg–Cd–Te detector cooled to 77 K; typically, 100 scans over the range 500–4500 cm^–1^ at a resolution of 0.25 cm^–1^ were acquired.

To record fluorescence spectra, the sample was irradiated with the output of an optical parametric oscillator (OPO, EKSPLA NT340) pumped by a frequency tripled Nd:YAG laser (EKSPLA NT300) operated at a repetition rate of 10 Hz. The OPO output beam was expanded to a diameter of ∼1.5 cm through a telescope to maximize the overlap with the sample. Emitted light was collected with a convex lens (*f* = 50 mm) and transmitted to the spectrograph via an optical fiber. The spectrograph consisted of a monochromator (Andor Shamrock SR500i, focal length 0.5 m) with a holographic grating (2400 grooves mm^–1^, reciprocal linear dispersion 0.83 nm mm^–1^), and an intensified charge-coupled device (iCCD, Andor iStar DH320T-18U-73, 1024 × 225 pixel, pixel size 26 μm × 26 μm). The entrance slit width of the monochromator was set to ∼25 μm for dispersed fluorescence recordings; in this configuration, each pixel of the iCCD corresponds to 0.022 nm, that is ∼1 cm^–1^ at a wavelength around 470 nm. To increase the throughput, the slit width was increased to 1.5–2.0 mm for the acquisition of lifetime and fluorescence excitation spectra. Emitted light was typically collected, starting 10 ns after excitation of the sample, for 75 ns. Absolute emission wavelengths were calibrated against a Hg(Ar) lamp; all dispersed fluorescence spectra have been corrected for wavelength-dependent variations in the sensitivity of the grating and the detector as specified by the manufacturer.

Fluorescence excitation spectra were obtained on stepping the OPO wavelength at increments of 0.1 nm and probing the recorded dispersed fluorescence spectra over a specific wavelength range. The line width of the OPO beam is <6 cm^–1^ in the range up to 400 nm and <4 cm^–1^ between 400 and 450 nm. Fluorescence excitation spectra displayed here have been corrected for variations in laser power with emission wavelength according to the specifications of the OPO system provided by the manufacturer.

Due to the extremely low vapor pressure of *peri*-HBC, preparation of premixed mixtures of the sample with *para*-H_2_ was challenging. Sample mixtures were therefore generated by passing a flow of *para*-H_2_ (flow rate ∼15 STP cm^3^ s^–1^) over a sample of solid *peri*-HBC heated to ∼300 °C. *para*-H_2_ was prepared from *normal*-H_2_ (Chiah-Lung, purity 99.9999%) by employing a Fe(III)-oxide catalyst cooled to ∼13 K; with this configuration, the minimum mole fraction of *ortho*-H_2_ attainable was estimated to be ∼100 ppm.

All quantum-chemical calculations were performed with the Gaussian 16 program package, Revision B.01.^32^ We conducted geometry optimizations and harmonic-frequency calculations for the electronic ground state and selected electronic excited states with two methods of (time-dependent) density functional theory, (TD-)B3PW91/6-311++G(2d,2p) and (TD-)wB97xD/6-311+G(d,p); in the latter functional, a dispersion correction to treat electrostatic interactions is implemented.^33^ Vibrationally resolved electronic absorption and emission spectra were simulated according to the optimized geometries and scaled harmonic vibrational frequencies of the electronic states involved by the Franck–Condon Herzberg–Teller approach. For comparison to our experimental results, we convoluted the computed stick spectra with a Gaussian line shape using a full width at half-maximum (fwhm) of 30 and 25 cm^–1^ for the simulated emission and absorption spectra, respectively; these parameters were chosen to match the line shapes observed in our experiments. Hamonic vibrational frequencies were scaled by empirical scaling factors of 0.982 (B3PW91) and 0.972 (wB97xD), respectively, to account for systematic deviations due to calculation errors and effects of the matrix environment; we determined these scaling factors in our earlier works^29,30^ by comparing the experimental IR spectra of a variety of planar neutral PAH isolated in solid *para*-H_2_ studied in our laboratory to those calculated with these two methods.

## 3 Results and Discussion

### 3.1 Dispersed Fluorescence Spectrum of *peri*-HBC

**Figure 2:**
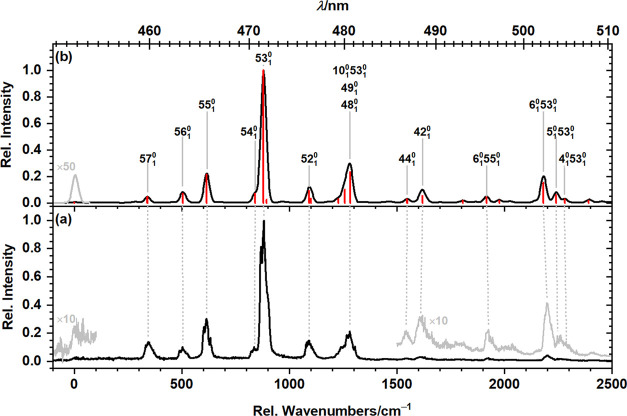
(a) Dispersed fluorescence spectrum of *peri*-HBC isolated in solid *para*-H_2_ recorded upon excitation at 372.7 nm (26,831 cm^–1^) and (b) S_1_ → S_0_ emission spectrum simulated at the (TD-)B3PW91/6-311++G(2d,2p) level; vibrational wavenumbers were scaled by 0.982. The top abscissa is wavelength (nm) and the bottom abscissa is wavenumbers (cm^–1^) below the origin at 452.7 nm (22,088 cm^–1^). The computed stick spectrum (red) was convoluted with a Gaussian line shape of fwhm 30 cm^–1^; transition mode numbers are indicated.

The dispersed fluorescence spectrum of *peri*-HBC isolated in solid *para*-H_2_ is depicted in Figure 2a. Emission was monitored upon excitation at 372.7 nm (26831 cm^–1^) corresponding to excitation to the S_4_(*E*_2u_) state. In matrix isolation spectroscopy, vibronic relaxation of excited states by internal conversion is typically fast, and, hence, emission typically occurs from the ground vibrational level of the lowest electronic excited state of the same spin multiplicity. The electronic transition origin, 0_0_^0^ band, can be located by comparing the dispersed fluorescence and the fluorescence excitation spectra as illustrated in Figure S1 and Table S1; it corresponds to a very weak feature observed at 452.7 nm (22088 cm^–1^). The spectral bands are sparse, with the most intense band located at 471.53 nm (880 cm^–1^ from the origin) and five notably less intense features at 459.95, 463.32, 465.67, 476.29, and 480.53 nm (347, 505, 614, 1093, and 1278 cm^–1^ from the origin).

*Peri*-HBC is a molecule of (approximate) *D*_6*h*_ symmetry with 116 vibrational normal modes, which can be represented by Γ_vib_ = 10 a_1g_ + 9 a_2g_ + 3 b_1g_ + 7 b_2g_ + 9 e_1g_ + 20 e_2g_ + 3 a_1u_ + 6 a_2u_ + 10 b_1u_ + 10 b_2u_ + 19 e_1u_ + 10 e_2u_, with vibrations of a_2u_ and e_1u_ symmetry being IR-active. Vibrational normal modes of a_2u_ symmetry represent out-of-plane deformations, and vibrational normal modes of e_1u_ symmetry correspond to mostly in-plane deformations asymmetric with respect to the inversion center. Vibrational wavenumbers for all normal modes of the S_0_ and the S_1_ states obtained with the wB97xD and B3PW91 methods are listed in Table S2. Rouillé et al.^21^ optimized the ground-state geometry of *peri*-HBC employing various combinations of functionals and basis sets and pointed out that optimizations strictly retaining *D*_6*h*_ symmetry were only successful at the B3LYP/6-311G level. Likewise, our calculations indicate an out-of-plane deformation of the molecule in its electronic ground state, and, consequently, degenerate vibrational normal modes of *e* symmetry are slightly split; the frequencies of *e* symmetric vibrations listed in Table S2 correspond to the averages.

Calculations at the TD-B3PW91/6-311++G(2d,2p) level predict a vertical excitation wavelength for the lowest excited singlet (S_1_) state of approximate B_2u_ symmetry of 450.81 nm (22,182 cm^–1^); S_2_(B_1u_) and S_3_(E_2g_) were predicted at 429.97 nm (23,257 cm^–1^) and 402.43 nm (24,849 cm^–1^), respectively; they all have an oscillator strength of 0.00. Considering that the ground electronic state is of symmetry A_1g_, the lowest-energy transition is symmetry-forbidden, consistent with the very weak 0_0_^0^ band observed at 22088 ± 12 cm^–1^ in our experiment. The first excited singlet state with a nonzero oscillator strength *f* is S_4_(E_1u_), predicted at 373.72 nm (26,758 cm^–1^) with *f* = 1.46. Vibrational modes contributing to the S_1_–S_0_ emission and absorption spectra should therefore gain intensities from interactions with the S_4_ state which is feasible only for normal modes of e_2g_ symmetry, in line with the conclusions drawn by Bräuchle.^18^

The observed spectrum is compared to the simulated S_1_ → S_0_ emission spectrum, calculated at the B3PW91/6-311++G(2d,2p) level of theory by a Franck–Condon Herzberg–Teller approach, in Figure 2. The computed stick spectrum (red) was convoluted with a Gaussian line shape of full width at half-maximum (fwhm) 30 cm^–1^ (black curve). Assignments for peaks in the dispersed fluorescence spectrum derived from a comparison of our experimental data to the simulated spectrum are provided in Figure 2b and listed in Table 1. The spectrum is exclusively composed of vibrational normal modes of approximate e_2g_ symmetry, and combination bands of the most intense vibrational mode ν_53_ (e_2g_, predicted at 880, 881 cm^–1^ in solid *para*-H_2_) with approximately total symmetric vibrational normal modes of a_1g_ symmetry. We therefore assigned the observed progression to the S_1_–S_0_ transition. Experimental and theoretically predicted peak positions are in good agreement with a mean absolute deviation of 4 ± 3 cm^–1^ for peaks contributed solely by fundamental normal modes.

**Table 1:** Peak Assignments in the Dispersed Fluorescence of *peri*-HBC Isolated in Solid *para*-H_2_

*para*-H_2_		B3PW91			wB97xD			
LIF		FCHT^a^	scaled^b^	int.^c^	scaled^d^	int.^c^		
nm	cm^–1^	cm^–1^	cm^–1^	%	cm^–1^	%	assignment	sym.
452.70	0	0	0	0.0	0	0.0	0_0_^0^	
459.95	347	342	340	4.3	342	2.9	57_1_^0^	e_2g_
463.32	505	503	504	6.8	504	3.2	56_1_^0^	e_2g_
		(554)^e^			554	5.2	(35_1_^0^)^e^	e_1g_
465.67	614	617	615	21.3	619	26.3	55_1_^0^	e_2g_
					621	2.3	(34_1_^0^)	e_1g_
					649	8.0	(29_1_^0^55_1_^0^)	
470.58	838	840	840	6.7	839	10.3	54_1_^0^	e_2g_
					869	2.8	(29_1_^0^54_1_^0^)	
471.53	881	880	880	100.0	879	100.0	53_1_^0^	e_2g_
					909	41.3	(29_1_^0^53_1_^0^)	
					939	2.5	(29_1_^0^53_1_^0^)	
476.29	1093	1092	1091	9.1	1091	15.3	52_1_^0^	e_2g_
					1103	6.1	(51_1_^0^)	e_2g_
					1121	3.8	(29_1_^0^52_1_^0^)	
480.53	1278	1281	1228	2.9	1225	2.3	10_1_^0^53_1_^0^	e_2g_
			1258	9.7	1250	13.8	49_1_^0^	e_2g_
			1282	23.0	1272	35.2	48_1_^0^	e_2g_
					1280	3.3	(29_1_^0^49_1_^0^)	
					1302	13.4	(29_1_^0^48_1_^0^)	
486.72	1543	1548	1548	2.3	1557	5.1	44_1_^0^	e_2g_
488.32	1610	1618	1619	5.2	1622	9.0	42_1_^0^ or 43_1_^0^^f^	e_2g_
					1641	3.0	(42_1_^0^)	e_2g_
					1652	3.6	(29_1_^0^43_1_^0^)	
495.92	1924	1917	1916	4.5	1924	8.6	6_1_^0^55_1_^0^	e_2g_
					1954	2.7	(6_1_^0^29_1_^0^55_1_^0^)	
		(2144)			2144	3.0	(5_1_^0^54_1_^0^)	e_2g_
502.73	2197	2180	2181	15.0	2184	44.1	6_1_^0^53_1_^0^	e_2g_
					2214	10.6	(6_1_^0^29_1_^0^53_1_^0^)	
504.30	2259	2239	2240	5.9	2251	10.4	5_1_^0^53_1_^0^	e_2g_
505.04	2288	2278	2281	2.7	2279	9.6	4_1_^0^53_1_^0^	e_2g_
					2281	4.0	(5_1_^0^29_1_^0^53_1_^0^)	
					2309	4.4	(4_1_^0^29_1_^0^53_1_^0^)	

a Peak positions in the convoluted stick spectrum using Gaussian shape with fwhm 30 cm^–1^.

b Harmonic vibrational wavenumbers scaled by 0.982.

c Relative intensities of the individual modes in the predicted stick spectrum in %; only modes with intensity ≥2.7% are included.

d Harmonic vibrational wavenumbers scaled by 0.972.

e Modes in parentheses are predicted to contribute to the spectrum only for calculations at the wB9xD/6-311+G(d,p) level.

f 42_1_^0^ according to B3PW91/6-311++G(2d,2p) and 43_1_^0^ according to wB97xD/6-311+G(d,p).

Intensities of peaks in vibrationally resolved electronic spectra are determined by the Franck–Condon factors of the contributing vibrational modes, and, thus, related to the changes in molecular geometry induced by the electronic transition and the vibrational motion. The S_1_–S_0_ electronic transition of *peri*-HBC can be described as a π–π* transition and is a superposition of the orbital transitions between HOMO – 1 and lowest unoccupied molecular orbital (LUMO), and highest occupied molecular orbital (HOMO) and LUMO + 1, respectively, which are depicted in Figure S2. As a result, changes in geometry upon transition between S_0_ and S_1_ impact mostly the C–C bond lengths, as depicted in Figure S3; the C–H bond lengths remain nearly unchanged and changes in bond angles are generally below 0.2 degrees.

**Figure 3:**
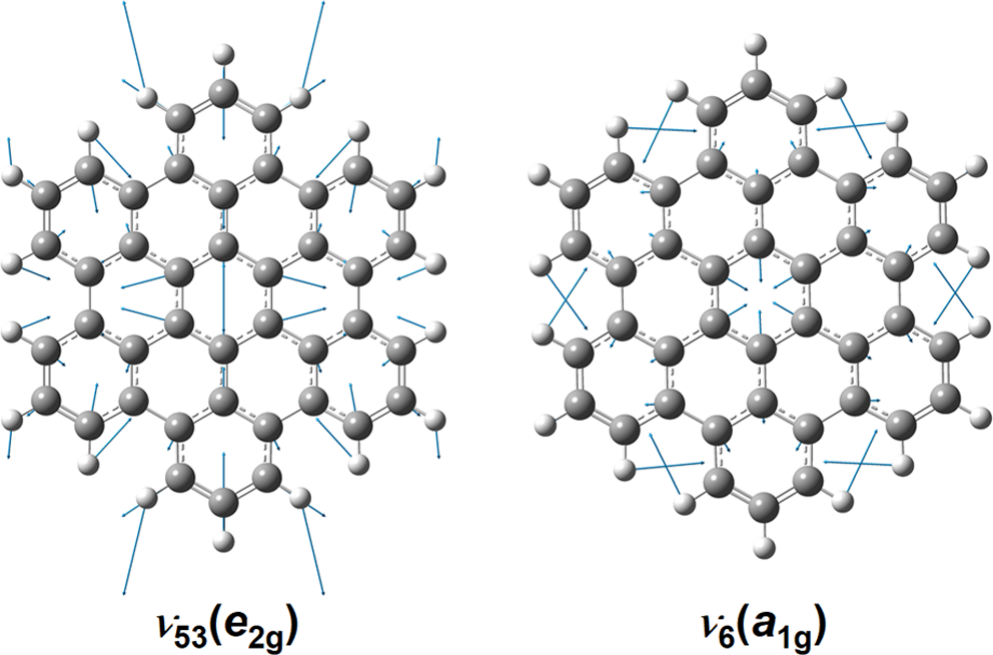
Displacement vectors of the vibrational normal modes ν_53_ (880 cm^–1^, e_2g_) and ν_6_ (1301 cm^–1^, a_1g_) of the S_0_ state of *peri*-HBC predicted at the B3PW91/6-311++G(2d,2p) level.

In their discussion of the aromaticity of nonplanar PAH, Antic et al.^34^ compared various methods to quantify the deviation from absolute planarity, including an approach based on dihedral angles proposed by Dobrowski et al.,^35^ who defined the ring planarity index *T* as
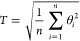
1where θ_*i*_ is the CCCC dihedral angle and *n* is the number of C atoms in the ring. The planarity index of the complete PAH equals the sum of the planarity indices of the individual rings. A planarity index of zero, hence, corresponds to perfect planarity and a planarity index of  to complete nonplanarity for all-benzenoid PAH consisting of *k* benzene rings. For *peri*-HBC optimized at the B3PW91/6-311++G(2d,2p) level of theory, eq 1 gives planarity indices of 22.6 and 25.7 for the S_0_ and S_1_ states, respectively, indicating that only a small additional out-of-plane deformation of the molecule is induced by the electronic transition. Consistently, only vibrational normal modes of e_2g_ and a_1g_ symmetry—these correspond to in-plane deformations of the molecule—contribute to the dispersed fluorescence spectrum; representations of ν_53_ (880 cm^–1^, e_2g_), the most intense vibrational mode, and ν_6_ (1301 cm^–1^, a_1g_), a main contributor to combination bands, are depicted in Figure 3. CH-stretching modes which alter only the C–H bond lengths do not appear in the spectrum. A comparison to the IR spectrum of *peri*-HBC in solid *para*-H_2_ is not feasible due to the different selection rules that apply.

A larger out-of-plane deformation associated with the S_1_–S_0_ transition is predicted at the (TD-)wB97xD/6-311+G(d,p) level of theory, with planarity indices of 29.7 and 38.4 for the S_0_ and S_1_ state, respectively. Simulated emission spectra calculated according to geometries optimized at the (TD-)B3PW91/6-311++G(2d,2p) and (TD-)wB97xD/6-311+(d,p) methods are compared in Figure S4 and give an impression of the impact of out-of-plane deformation on the electronic spectra of *peri*-HBC: simulations at the (TD-)wB97xD/6-311+G(d,p) level predict a non-negligible contribution of combination bands of e_2g_ vibrational normal modes with ν_29_ (30 cm^–1^, b_2g_), cf. Table 1. This mode, depicted in Figure S5, corresponds to an out-of-plane motion of the C–H moieties in alternating directions from one outer ring to the next, and resembles the out-of-plane deformation induced by the electronic transition. However, we did not observe clear evidence of these types of combination bands.

### 3.2 Fluorescence Excitation Spectrum of *peri*-HBC

**Figure 4:**
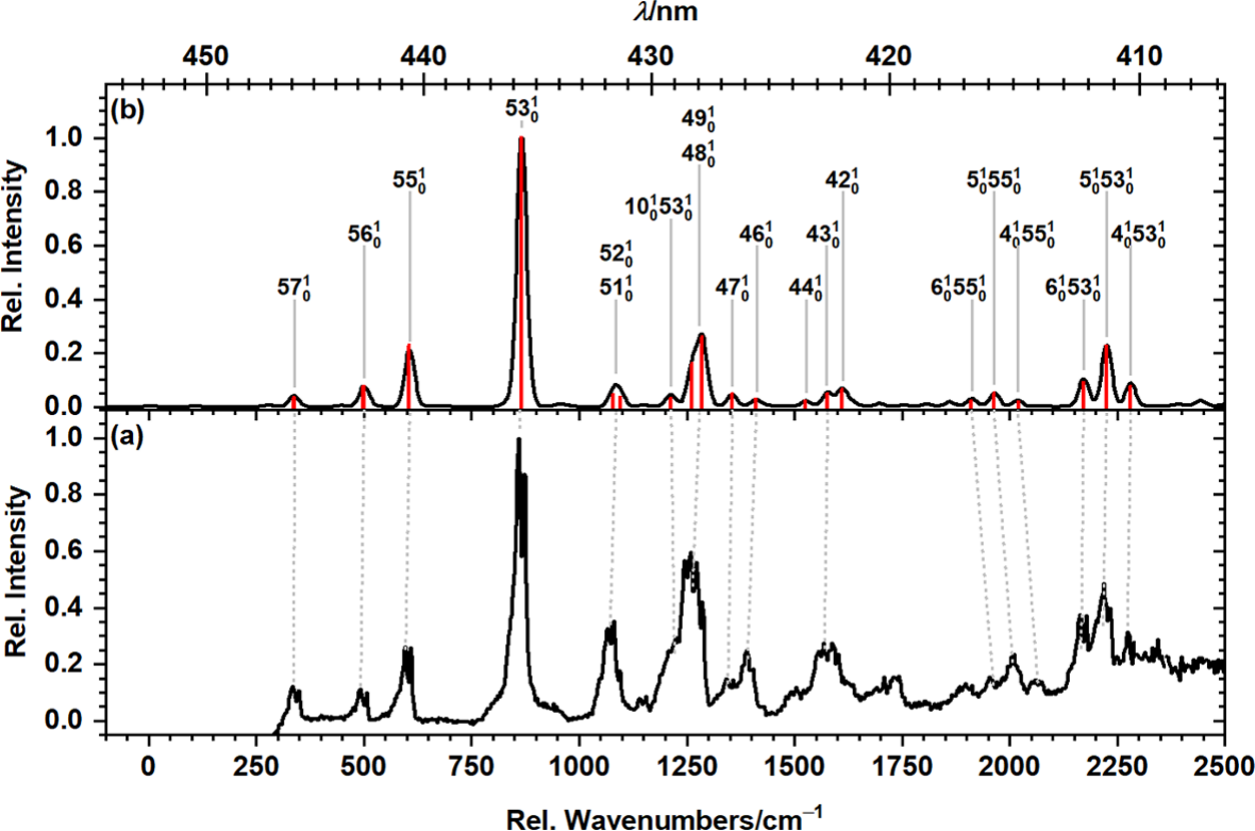
(a) Fluorescence excitation spectrum of *peri*-HBC isolated in solid *para*-H_2_ recorded by probing emission in the range 21,155–21,254 cm^–1^ and (b) S_1_ ← S_0_ absorption spectrum simulated at (TD-)B3PW91/6-311++G(2d,2p) level. Vibrational wavenumbers were scaled by 0.982. The top abscissa is wavelength (nm) and the bottom abscissa is wavenumbers (cm^–1^) above the origin at 452.7 nm (22,088 cm^–1^). The computed stick spectrum (red) was convoluted with a Gaussian line shape of fwhm 25 cm^–1^; transition mode numbers are indicated.

The fluorescence excitation spectrum of *peri*-HBC isolated in *para*-H_2_ obtained on probing fluorescence emission in the range 470.5–472.7 nm (21,155–21,254 cm^–1^), i.e., the most intense feature in the dispersed fluorescence spectrum corresponding to 53_1_^0^, is depicted in Figure 4a and compared to the simulated absorption spectrum obtained from calculations with the B3PW91/6-311++G(2d,2p) method. The computed stick spectrum (red) was convoluted with a Gaussian line shape of fwhm 25 cm^–1^ (black curve). Peak positions and relative intensities from experiments and calculations agree satisfactorily, with the most intense peak observed in the experimental spectrum at 435.7 nm (862 cm^–1^ above the experimental origin) and predicted at 866 cm^–1^ above the predicted origin; the mean absolute deviation between peak positions in the simulated and the experimental spectrum is 8 ± 6 cm^–1^, slightly larger than for the dispersed fluorescence spectrum but appears reasonable considering the uncertainties induced by applying the scaling factor of vibrational wavenumbers of the ground electronic state to those of the excited electronic state. The larger peak intensities and additional features observed in regions >1000 cm^–1^ above the origin might contain contributions of the S_2_ state which was predicted ∼1075 cm^–1^ above S_1_, within the spectral range depicted in Figure 4. The simulated S_2_ ← S_0_ absorption spectrum of *peri*-HBC is depicted in Figure S6; it consists of an intense peak 951 cm^–1^ above the extremely weak 0_0_^0^ origin (relative intensity ∼2%) and, among other weak features, two bands with relative intensities >20% at 204 and 2310 cm^–1^ above the 0_0_^0^ band. Our simulations predict maximum intensities for the S_1_ and S_2_ absorption spectra of 22,340.2 and 536,748 dm^3^ mol^–1^ cm^–1^, respectively. The combined spectra of the S_1_ and S_2_ absorption assuming an energy difference between the two states of 775 and 1205 cm^–1^, respectively, are illustrated in Figure S7. Both combinations improve the agreement between the simulated and the experimental spectra either by additional peaks or by changes in the relative intensities; nonetheless, locating the definitive position of the S_2_ state in solid *para*-H_2_ from our fluorescence excitation spectrum is difficult at this stage.

Most spectral features in the fluorescence excitation spectrum exhibit a splitting of ∼11 cm^–1^, likely due to the occupation of different positions in the matrix lattice by the guest molecules, as was previously observed for the 1-C_9_H_10_ radical in solid *para*-H_2_.^30^ We therefore determined peak positions in the fluorescence excitation spectrum by fitting the spectrum with a set of Gaussians after smoothening the spectrum by applying a window of ∼10 cm^–1^. Partial dispersed fluorescence spectra obtained after excitation at the two different components of one peak in the excitation spectra are displayed in Figure S8 and exhibit the same relative shift.

Similar to the dispersed fluorescence spectrum, the fluorescence excitation spectrum is composed exclusively of vibrational modes of e_2g_ symmetry and their combination bands with vibrations of totally symmetric a_1g_. Assignments for the observed features in the excitation spectrum derived from a comparison to the simulated absorption spectrum are listed in Table 2; all vibrations associated with the S_1_ state of *peri*-HBC calculated at the TD-B3PW91/6-311++G(2d,2p) level are provided in Table S2.

**Table 2:** Peak Assignments in the Fluorescence Excitation Spectrum of *peri*-HBC Isolated in Solid *para*-H_2_

*para*-H_2_		B3PW91				
LIF		FCHT^a^	scaled^b^	int.^c^		
nm	cm^–1^	cm^–1^	cm^–1^	%	assignment	sym.
452.7	0	0	0	0.0	0_0_^0^	
445.9	338	338	337	4.3	57_0_^1^	e_2g_
442.9	492	500	499	7.8	56_0_^1^	e_2g_
440.8	597	606	605	23.1	55_0_^1^	e_2g_
435.7	862	866	866	100.0	53_0_^1^	e_2g_
431.8	1071	1086	1080	4.7	52_0_^1^	e_2g_
			1095	3.4	51_0_^1^	e_2g_
429.0	1220	1213	1213	3.3	10_0_^1^53_0_^1^	e_2g_
428.2	1263	1279	1262	15.9	49_0_^1^	e_2g_
			1286	26.1	48_0_^1^	e_2g_
426.7	1345	1356	1355	4.9	47_0_^1^	e_2g_
425.9	1391	1412	1411	2.7	46_0_^1^	e_2g_
		1527	1526	2.1	44_0_^1^	e_2g_
422.7	1570	1575	1577	4.9	43_0_^1^	e_2g_
		1612	1611	6.5	42_0_^1^	e_2g_
					46_0_^1^	e_2g_
415.6	1972	1912	1910	2.2	6_0_^1^55_0_^1^	e_2g_
415.0	2007	1965	1964	5.2	5_0_^1^55_0_^1^	e_2g_
414.0	2065	2018	2020	1.9	4_0_^1^55_0_^1^	e_2g_
412.3	2167	2172	2171	8.7	6_0_^1^53_0_^1^	e_2g_
411.4	2217	2225	2224	22.7	5_0_^1^53_0_^1^	e_2g_
410.5	2274	2281	2281	7.7	4_0_^1^53_0_^1^	e_2g_

a Peak positions in the convoluted stick spectrum using Gaussian shape with fwhm 25 cm^–1^.

b Harmonic vibrational wavenumbers scaled by 0.982.

c Relative intensities of individual modes in the stick spectrum in %; only modes with intensity ≥1.9% are included.

No position for the forbidden 0_0_^0^ band of S_1_ ← S_0_ of gaseous *peri*-HBC has been previously reported in the literature. To assess the influence of *para*-H_2_ as a matrix host on the electronic spectrum of *peri*-HBC, we therefore compare the reported spectra directly. Kokkin et al.^22^ recorded the 2C2PI spectrum of jet-cooled *peri*-HBC over the range 408–453 nm and located the two most intense peaks at 433.52 nm (23,067 cm^–1^) and 426.41 nm (23,452 cm^–1^), respectively, with an intensity ratio of approximately 1:3. The peak at 23067 cm^–1^ likely corresponds to the absorption band 53_0_^1^, located 862 cm^–1^ above the 0_0_^0^ band, i.e., at 22,950 cm^–1^, implying a matrix shift of 117 cm^–1^. The high-resolution peak at 23,452 cm^–1^ of gaseous *peri*-HBC likely corresponds to the absorption band 48_0_^1^, but the observed band in *para*-H_2_ located 1263 cm^–1^ above the 0_0_^0^ band, i.e., at 23351 cm^–1^, includes bands 49_0_^1^ and 48_0_^1^. If we use the difference between the peak of 48_0_^1^ (1286 cm^–1^) and the convoluted peak position of 49_0_^1^ and 48_0_^1^ (1279 cm^–1^) to correct for the difference, we obtain a matrix shift of 94 cm^–1^. We hence estimate that the matrix shift of the transition origin is ∼106 ± 11 cm^–1^ from the average of these two values.

Rouillé et al.^21^ report three peaks in the absorption spectrum of *peri*-HBC in solid Ar at 424.00 (23,585 cm^–1^), 430.75 (23,215 cm^–1^), and 438.25 nm (22,818 cm^–1^); the latter, most intense feature, was also observed in solid Ne at 434.40 nm (23,020 cm^–1^). Under the assumption that the most intense feature corresponds to 53_0_^1^, matrix shifts of +47 and +249 cm^–1^ relative to the gas-phase value can be deduced for solid Ne and solid Ar, respectively. Our work thus suggests an impact of the *para*-H_2_ environment on the S_1_–S_0_ transition of *peri*-HBC smaller than for solid Ar but larger than for solid Ne as a matrix host, in line with our earlier study of the smaller, bowl-shaped PAH sumanene (C_21_H_12_).^29^ The relative intensities of the two absorption features discussed here are inversed between the 2C2PI study of Kokkin et al.^22^ and the matrix isolation studies; the relative intensities in our fluorescence excitation spectrum of *peri*-HBC are consistent with those in the absorption spectra in solid Ne and Ar presented by Rouillé et al.^21^ The variations in efficiencies of ionization upon excitation to various vibronic levels in 2C2PI study might be the reason for this discrepancy in intensity.

The most intense band in the S_1_ ← S_0_ fluorescence excitation spectrum of *peri*-HBC in *para-*H_2_ is located at 22,950 cm^–1^ or 435.7 nm, corresponding to the peak at 23,067 cm^–1^ or 433.52 nm in the gas phase. The DIB density in the 400–450 nm region is low; in their survey of DIB toward HD204827, Hobbs et al.^36^ identified three DIB in this range, located at 425.901, 436.386, and 442.819 nm with fwhm of 0.105, 0.046, and 2.25 nm, respectively. A contribution of *peri*-HBC to the DIB hence remains unlikely considering the significant deviation in wavelength and the extremely low oscillator strength of the forbidden S_1_–S_0_ transition; the first symmetry-allowed transition for *peri*-HBC, the S_4_–S_0_ transition predicted at ∼373 nm, lies outside the wavelength range where DIB are commonly observed.

## 4 Conclusions

We presented the first full vibronic analysis of the dispersed fluorescence and fluorescence excitation spectra associated with the symmetry-forbidden S_1_–S_0_ transition of *peri*-HBC. We recorded the spectra by *para*-H_2_ matrix isolation spectroscopy and analyzed them based on simulated spectra obtained from Franck–Condon Herzberg–Teller calculations according to optimized geometries and scaled harmonic vibrational wavenumbers calculated at the (TD-)B3PW91/6-311++G(2d,2p) level of theory. Theoretical predictions agree satisfactorily with the experimental spectra with average absolute deviations in peak positions ≤8 cm^–1^; observed features can be assigned exclusively to vibrational normal modes of e_2g_ symmetry and a few of their combination bands with totally symmetric (a_1g_) modes, consistent with selection rules for intensity borrowing from the next higher excited state S_4_ with a nonzero oscillator strength. We clearly observed the position of the S_1_–S_0_ electronic transition origin of *peri*-HBC at 22,088 cm^–1^ (452.7 nm), which was unreported previously. From a comparison of two corresponding features with the reported 2C2PI spectrum of jet-cooled *peri*-HBC by Kokkin et al.,^22^ we inferred a red shift of ∼110 cm^–1^ induced by the solid *para*-H_2_ environment; the shift induced by *para*-H_2_ is, hence, larger than the one observed for Ne but smaller than the one for Ar as a matrix host,^21^ consistent with our earlier studies. Although *peri*-HBC might be large enough to survive the interstellar UV radiation field, a contribution of *peri*-HBC to the DIB is rather unlikely because of the significant deviation from the reported wavelengths of DIB, the weakness of the S_1_–S_0_ electronic transitions, and the lack of reported DIB at <400 nm where the intense S_4_ ← S_0_ band of *peri*-HBC is located.

## Supporting Information Available

Determination of the position of the 0_0_^0^ band position of the S_1_–S_0_ transition in solid *para*-H_2_; calculated harmonic vibrational wavenumbers (cm^–1^) for the electronic ground state and the first excited electronic state of *peri*-HBC; molecular orbitals involved in the S_1_–S_0_ transition; representative bond length (Å) of *peri*-HBC in the S_0_ and S_1_ states; simulated S_1_ → S_0_ emission spectrum at two different levels of theory; displacement vectors associated with ν_29_; simulated S_2_ ← S_0_ absorption spectrum; comparison of the simulated combined S_2_ and S_1_ absorption spectrum to the experimental data; and partial dispersed fluorescence spectra on excitation at two different component of the same peak in the excitation spectrum (PDF)
