# Supplementary material for: Fluorescence Excitation and Dispersed Fluorescence Spectra of the First Electronic Excited (S1) State of peri-Hexabenzocoronene (C42H18) Isolated in Solid para-Hydrogen
Source: J Phys Chem A. 2024 Jun 12;128(25):4984–91. doi: 10.1021/acs.jpca.4c02320 (PMC11215764; doi:10.1021/acs.jpca.4c02320)
Supplement: Supplementary file 1 — jp4c02320_si_001.pdf [file jp4c02320_si_001.pdf]

Supporting Information

Fluorescence Excitation and Dispersed Fluorescence Spectra of the First Electronic Excited (S_1) State of *peri*-Hexabenzocoronene ($C_{42}H_{18}$) Isolated in Solid *Para*-Hydrogen

Isabelle Weber[†]* *and Yuan-Pern Lee*[†,‡]*

[†] Department of Applied Chemistry and Institute of Molecular Science, National Yang Ming Chiao Tung University, Hsinchu 3000093, Taiwan

[‡] Center for Emergent Functional Matter Science, National Yang Ming Chiao Tung University, Hsinchu 300093, Taiwan

Table of Contents

Table S1. Support for the location of the 0_0^0 band of the S_1–S_0 transition at 22088 cm^{-1}.......S3

Table S2. Vibrational wavenumbers of the ground (S_0) and the first excited (S_1) state of *peri*-HBC calculated at two DFT levels. S4

Figure S1. Dispersed fluorescence and fluorescence excitation spectra of *peri*-HBC in solid *para*-H_2 and mode assignments. S7

Figure S2. Molecular orbitals involved in the S_1–S_0 electronic transition of *peri*-HBC.........S8

Figure S3. Bond length (in Å) of the optimized geometries of the S_0 and S_1 states of *peri*-HBC predicted at the (TD-)B3PW91/6-311++G(2d,2p) level...S9

Figure S4. Normalized $S_1 \rightarrow S_0$ emission spectra simulated according to geometries optimized at the (TD-)B3PW91/6-311++G(2d,2p) and the (TD-)wB97xD/6-311+G(d,p) levels of theory. S10

Figure S5. Displacement vectors associated with mode ν_{29} (30 cm^{-1}, b_{2g}) of *peri*-HBC as predicted at the wB97xD/6-311+G(d,p) level of theory...S11

Figure S6. Simulated electronic absorption spectrum corresponding to the $S_2 \leftarrow S_0$ transition of *peri*-HBC at the TD-B3PW91/6-311++G(2d,2p) level of theory.......................................S122

Figure S7. Comparison of experimental fluorescence excitation spectrum of *peri*-HBC isolated in solid *para*-H_2 with simulations...S133

Figure S9. Partial dispersed fluorescence spectra of *peri*-HBC in solid *para*-H_2 after excitation at 435.3 nm and 435.5 nm...S144

Table S1. Support for the Location of the 0_0^0 Band of the S_1–S_0 Transition at 22088 cm^{-1}. Centers Among Four Pairs of Peaks near the 0_0^0 Band in the Dispersed Fluorescence and the Fluorescence Excitation Spectra of *Peri*-HBC in Solid *Para*-H_2.

mode	$S_1 \leftarrow S_0$	center	$S_1 \rightarrow S_0$
	cm^{-1}	cm^{-1}	cm^{-1}
ν_{57}	22435	22088.5	21742
ν_{56}	22593	22088.5	21584
ν_{55}	22697	22086.0	21475
ν_{53}	22961	22084.5	21208

Table S2. Vibrational Wavenumbers of the Ground (S_0) and the First Excited (S_1) State of *Peri*-HBC Calculated at Two DFT Levels.

mode	symmetry	S_0				S_1
		wB97xD		B3PW91		B3PW91
		scaled[a]	IR int.[b]	scaled[c]	IR int.[b]	scaled[c]
ν_1	(a_{1g})	3159	(0)	3177	(0)	3178
ν_2	(a_{1g})	3111	(0)	3130	(0)	3131
ν_3	(a_{1g})	1627	(0)	1613	(0)	1573
ν_4	(a_{1g})	1399	(0)	1401	(0)	1415
ν_5	(a_{1g})	1372	(0)	1360	(0)	1359
ν_6	(a_{1g})	1305	(0)	1301	(0)	1305
ν_7	(a_{1g})	1162	(0)	1154	(0)	1146
ν_8	(a_{1g})	1008	(0)	1006	(0)	985
ν_9	(a_{1g})	732	(0)	731	(0)	722
ν_{10}	(a_{1g})	346	(0)	348	(0)	347
ν_{11}	(a_{2g})	3133	(0)	3155	(0)	3156
ν_{12}	(a_{2g})	1623	(0)	1610	(0)	1577
ν_{13}	(a_{2g})	1459	(0)	1461	(0)	1450
ν_{14}	(a_{2g})	1337	(0)	1355	(0)	1348
ν_{15}	(a_{2g})	1236	(0)	1235	(0)	1230
ν_{16}	(a_{2g})	1165	(0)	1163	(0)	1159
ν_{17}	(a_{2g})	780	(0)	782	(0)	781
ν_{18}	(a_{2g})	617	(0)	614	(0)	607
ν_{19}	(a_{2g})	506	(0)	501	(0)	501
ν_{20}	(b_{1g})	905	(0)	897	(0)	882
ν_{21}	(b_{1g})	461	(0)	465	(0)	455
ν_{22}	(b_{1g})	102	(0)	102	(0)	103
ν_{23}	(b_{2g})	1146	(0)	1051	(0)	1039
ν_{24}	(b_{2g})	969	(0)	962	(0)	950
ν_{25}	(b_{2g})	801	(0)	814	(0)	803
ν_{26}	(b_{2g})	695	(0)	698	(0)	687
ν_{27}	(b_{2g})	505	(0)	502	(0)	485
ν_{28}	(b_{2g})	355	(0)	354	(0)	339
ν_{29}	(b_{2g})	30	(0)	21	(0)	19
ν_{30}	(e_{1g})	978	(0)	971	(0)	959
ν_{31}	(e_{1g})	894	(0)	889	(0)	874
ν_{32}	(e_{1g})	785	(0)	793	(0)	781
ν_{33}	(e_{1g})	736	(0)	745	(8)	730
ν_{34}	(e_{1g})	621	(0)	627	(0)	618
ν_{35}	(e_{1g})	554	(0)	552	(0)	533
ν_{36}	(e_{1g})	379	(0)	376	(0)	366
ν_{37}	(e_{1g})	209	(0)	207	(0)	200
ν_{38}	(e_{1g})	115	(0)	114	(0)	112
ν_{39}	(e_{2g})	3157	(0)	3174	(1)	3175

ν_{40}	(e_{2g})	3138	(0)	3160	(0)	3160
ν_{41}	(e_{2g})	3108	(0)	3128	(0)	3129
ν_{42}	(e_{2g})	1641	(0)	1619	(0)	1611
ν_{43}	(e_{2g})	1622	(0)	1605	(0)	1577
ν_{44}	(e_{2g})	1557	(0)	1548	(0)	1526
ν_{45}	(e_{2g})	1490	(0)	1473	(0)	1440
ν_{46}	(e_{2g})	1457	(0)	1455	(0)	1411
ν_{47}	(e_{2g})	1342	(0)	1353	(0)	1355
ν_{48}	(e_{2g})	1272	(0)	1282	(0)	1286
ν_{49}	(e_{2g})	1250	(0)	1258	(0)	1262
ν_{50}	(e_{2g})	1213	(0)	1208	(0)	1203
ν_{51}	(e_{2g})	1103	(0)	1100	(0)	1095
ν_{52}	(e_{2g})	1091	(0)	1091	(0)	1080
ν_{53}	(e_{2g})	879	(0)	880	(0)	866
ν_{54}	(e_{2g})	839	(0)	840	(0)	830
ν_{55}	(e_{2g})	619	(0)	615	(0)	605
ν_{56}	(e_{2g})	504	(0)	504	(0)	499
ν_{57}	(e_{2g})	342	(0)	340	(0)	337
ν_{58}	(e_{2g})	275	(0)	274	(0)	272
ν_{59}	(a_{1u})	891	(0)	887	(0)	872
ν_{60}	(a_{1u})	664	(0)	675	(0)	665
ν_{61}	(a_{1u})	177	(0)	174	(0)	165
ν_{62}	(a_{2u})	980	(0)	974	(0)	961
ν_{63}	(a_{2u})	773	(100)	769	(100)	757
ν_{64}	(a_{2u})	721	(4)	743	(12)	732
ν_{65}	(a_{2u})	524	(0)	526	(1)	513
ν_{66}	(a_{2u})	279	(2)	276	(2)	262
ν_{67}	(a_{2u})	81	(2)	81	(2)	80
ν_{68}	(b_{1u})	3140	(0)	3161	(0)	3162
ν_{69}	(b_{1u})	3107	(0)	3127	(0)	3129
ν_{70}	(b_{1u})	1645	(0)	1629	(0)	1590
ν_{71}	(b_{1u})	1513	(0)	1512	(0)	1516
ν_{72}	(b_{1u})	1373	(0)	1381	(0)	1390
ν_{73}	(b_{1u})	1105	(0)	1100	(0)	1095
ν_{74}	(b_{1u})	1016	(0)	1015	(0)	1006
ν_{75}	(b_{1u})	998	(0)	1000	(0)	991
ν_{76}	(b_{1u})	668	(0)	665	(0)	661
ν_{77}	(b_{1u})	392	(0)	393	(0)	389
ν_{78}	(b_{2u})	3156	(0)	3174	(2)	3174
ν_{79}	(b_{2u})	1582	(0)	1561	(0)	1536
ν_{80}	(b_{2u})	1512	(0)	1507	(0)	1505
ν_{81}	(b_{2u})	1376	(0)	1386	(0)	1382
ν_{82}	(b_{2u})	1306	(0)	1317	(0)	1354
ν_{83}	(b_{2u})	1219	(0)	1244	(0)	1265
ν_{84}	(b_{2u})	1193	(0)	1205	(0)	1202
ν_{85}	(b_{2u})	933	(0)	936	(0)	936

ν_{86}	(b_{2u})	435	(0)	436	(0)	436
ν_{87}	(b_{2u})	351	(0)	347	(0)	346
ν_{88}	(e_{1u})	3158	(19)	3176	(23)	3176
ν_{89}	(e_{1u})	3135	(0)	3157	(0)	3157
ν_{90}	(e_{1u})	3110	(15)	3129	(18)	3130
ν_{91}	(e_{1u})	1636	(0)	1617	(1)	1581
ν_{92}	(e_{1u})	1615	(9)	1605	(11)	1551
ν_{93}	(e_{1u})	1504	(2)	1502	(3)	1490
ν_{94}	(e_{1u})	1419	(2)	1409	(1)	1403
ν_{95}	(e_{1u})	1384	(21)	1382	(25)	1368
ν_{96}	(e_{1u})	1348	(0)	1356	(0)	1335
ν_{97}	(e_{1u})	1308	(1)	1317	(1)	1266
ν_{98}	(e_{1u})	1233	(3)	1228	(4)	1199
ν_{99}	(e_{1u})	1166	(0)	1164	(0)	1160
ν_{100}	(e_{1u})	1146	(1)	1140	(1)	1126
ν_{101}	(e_{1u})	1102	(7)	1103	(11)	1081
ν_{102}	(e_{1u})	814	(0)	816	(0)	813
ν_{103}	(e_{1u})	681	(2)	681	(3)	671
ν_{104}	(e_{1u})	594	(2)	593	(3)	589
ν_{105}	(e_{1u})	539	(1)	547	(0)	545
ν_{106}	(e_{1u})	265	(0)	266	(0)	262
ν_{107}	(e_{2u})	973	(0)	965	(0)	953
ν_{108}	(e_{2u})	902	(0)	894	(0)	879
ν_{109}	(e_{2u})	867	(0)	819	(0)	806
ν_{110}	(e_{2u})	775	(1)	735	(0)	719
ν_{111}	(e_{2u})	653	(0)	636	(0)	616
ν_{112}	(e_{2u})	550	(0)	547	(0)	535
ν_{113}	(e_{2u})	457	(0)	456	(0)	448
ν_{114}	(e_{2u})	297	(0)	294	(0)	282
ν_{115}	(e_{2u})	113	(0)	110	(0)	108
ν_{116}	(e_{2u})	13	(0)	9	(0)	10

[a]Harmonic vibrational wavenumbers calculated at the wB97xD/6-311+G(d,p) level and scaled by 0.972. [b]Relative IR intensities in % of the most intense band (ν_{63}). [c]Harmonic vibrational wavenumbers calculated at the B3PW91/6-311++G(2d,2p) level and scaled by 0.982.

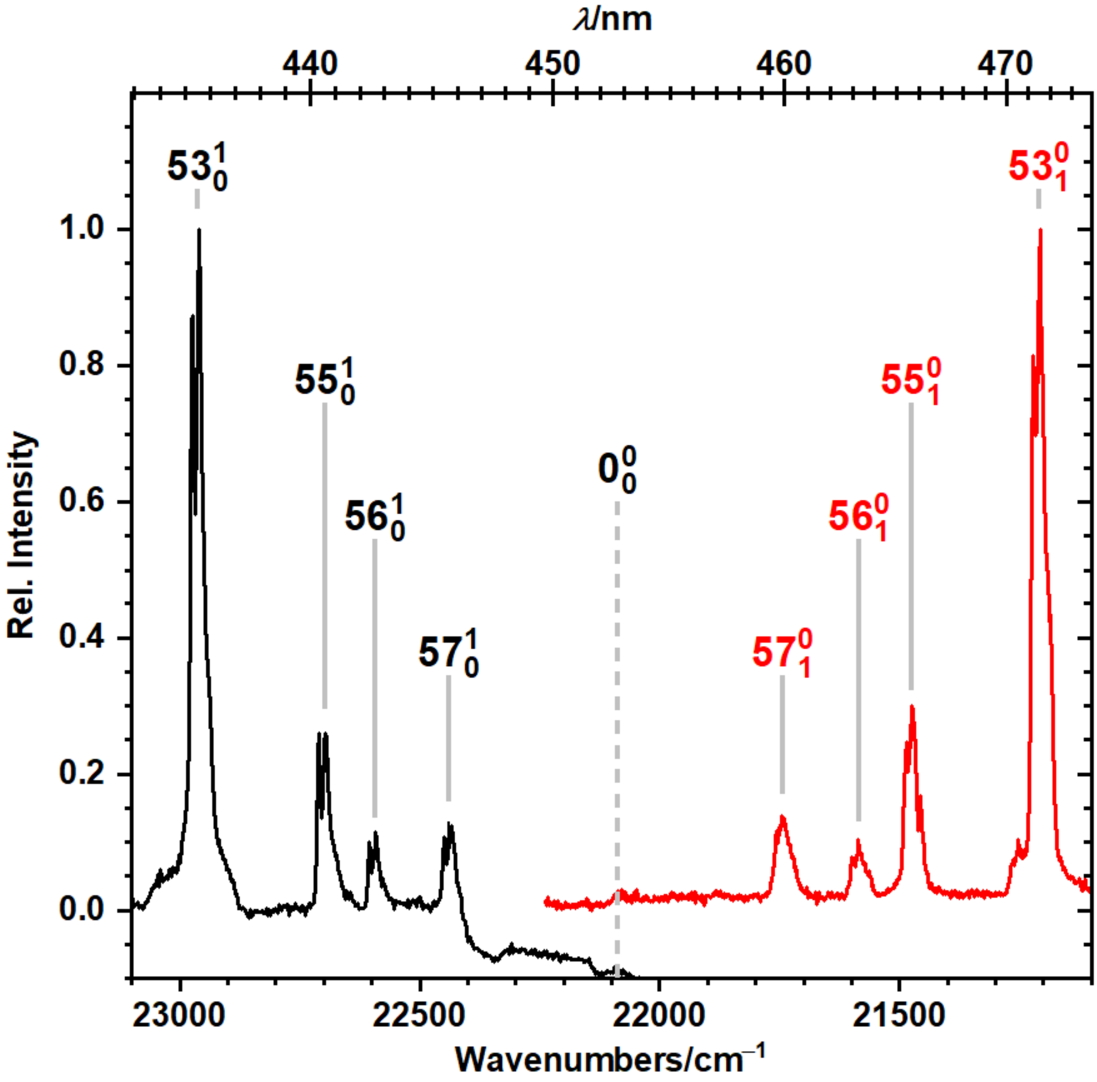

Figure S1. Dispersed fluorescence (red) and fluorescence excitation (black) spectra of *peri*-HBC in solid *para*-H_2 and mode assignments.

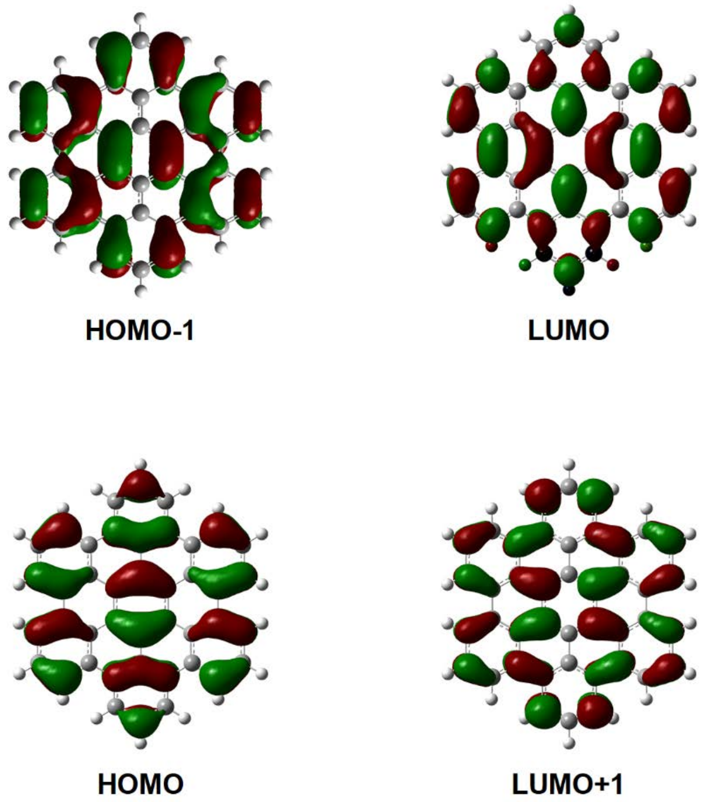

Figure S2. Molecular orbitals involved in the S_1–S_0 electronic transition of *peri*-HBC. All contours have been plotted with an isovalue of 0.02.

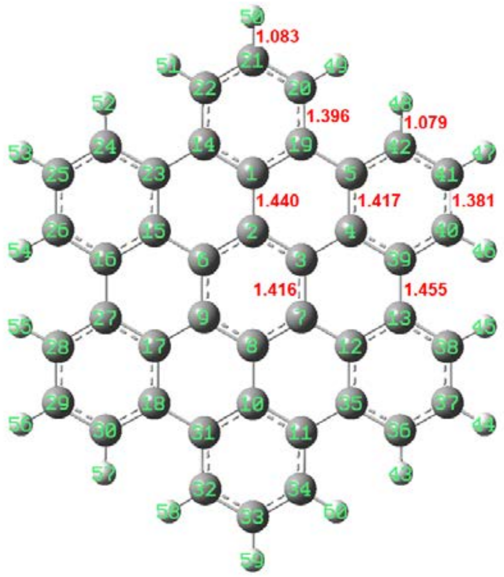

S_0

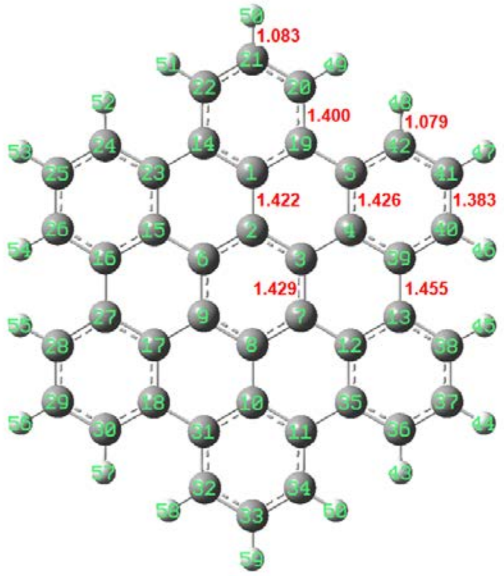

S_1

Figure S3. Bond length (in Å) of the optimized geometries of the S_0 and the S_1 states of *peri*-HBC predicted at the (TD-)B3PW91/6-311++G(2d,2p) level.

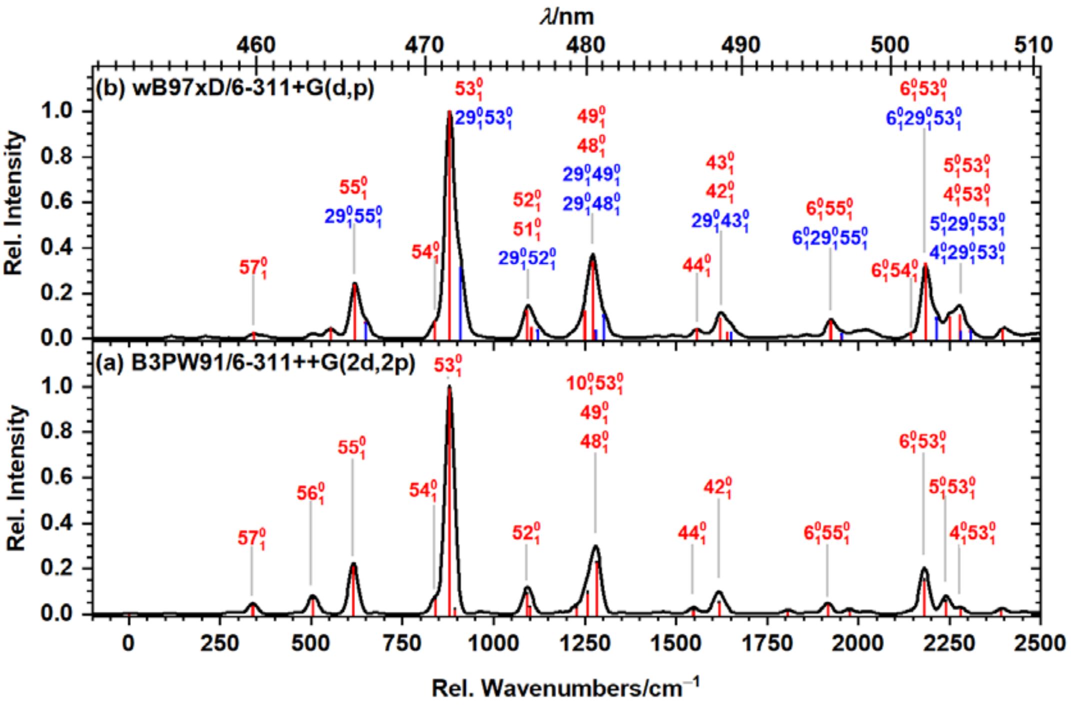

Figure S4. Normalized $S_1 \rightarrow S_0$ emission spectra simulated according to geometries optimized at the (TD-)B3PW91/6-311++G(2d,2p) (a) and the (TD-)wB97xD/6-311+G(d,p) (b) levels of theory. Vibrational wavenumbers have been scaled by 0.982 (B3PW91) and 0.972 (wB97xD), respectively. The normalized stick spectrum is shown in red. Blue sticks in panel (b) represent combination bands with the mode ν_{29} (30 cm^{-1}, b_{2g}). The computed stick spectrum (red) was convoluted with a Gaussian line shape of full-width-half-maximum (FWHM) 30 cm^{-1}. The top abscissa is wavelength (nm) and the bottom abscissa is wavenumbers (cm^{-1}) below the origin at 452.7 nm (22088 cm^{-1}).

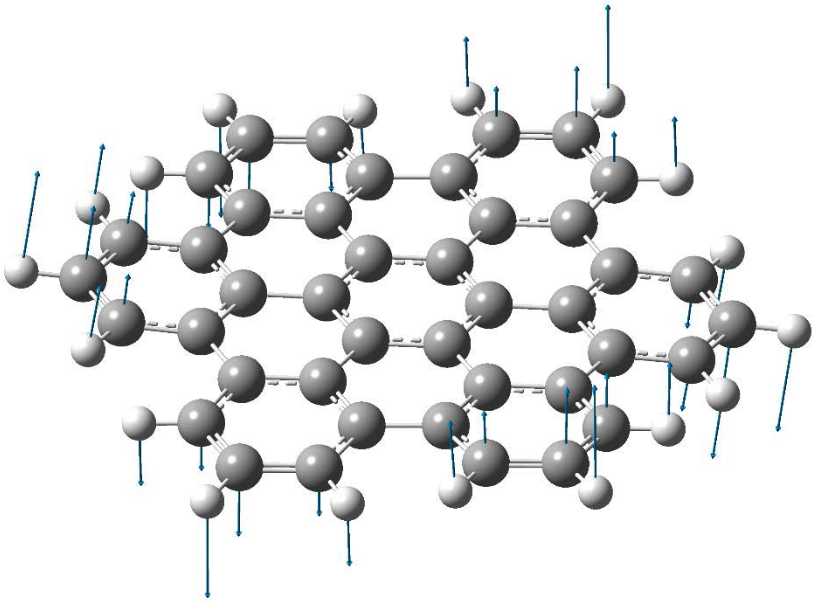

Figure S5. Displacement vectors associated with mode ν_{29} (30 cm^{-1}, b_{2g}) of *peri*-HBC as predicted at the wB97xD/6-311+G(d,p) level of theory.

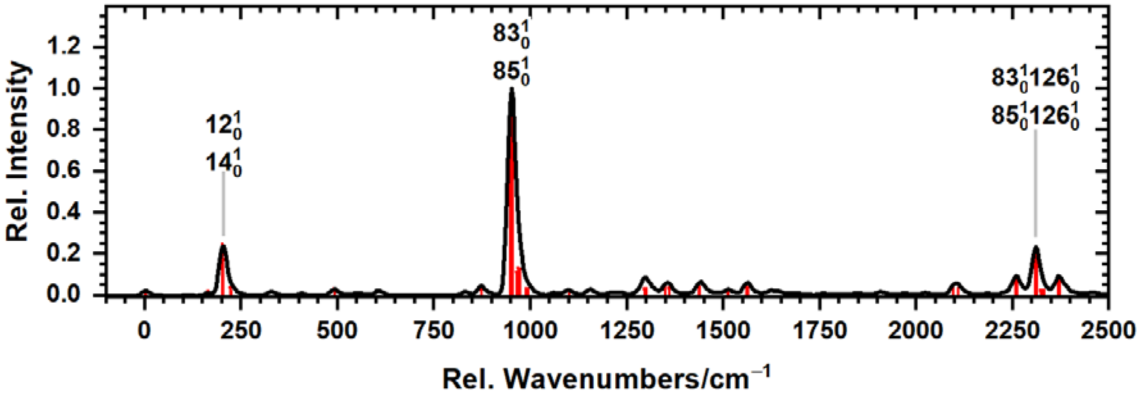

Figure S6. Simulated electronic absorption spectrum corresponding to the S_2←S_0 transition of *peri*-HBC at the TD-B3PW91/6-311++G(2d,2p) level of theory. Vibrational wavenumbers were scaled by 0.982. The normalized stick spectrum, which includes only vibrations with a relative intensity ≥2 % of the most intense band, is shown in red. The stick spectrum (red) was convoluted with a Gaussian line shape of full-width-at-half-maximum (FWHM) 25 cm^{-1}; transition mode numbers were indicated. The top abscissa is wavelength (nm) and the bottom abscissa is wavenumbers (cm^{-1}) above the origin of S_1←S_0 at 452.7 nm (22088 cm^{-1}).

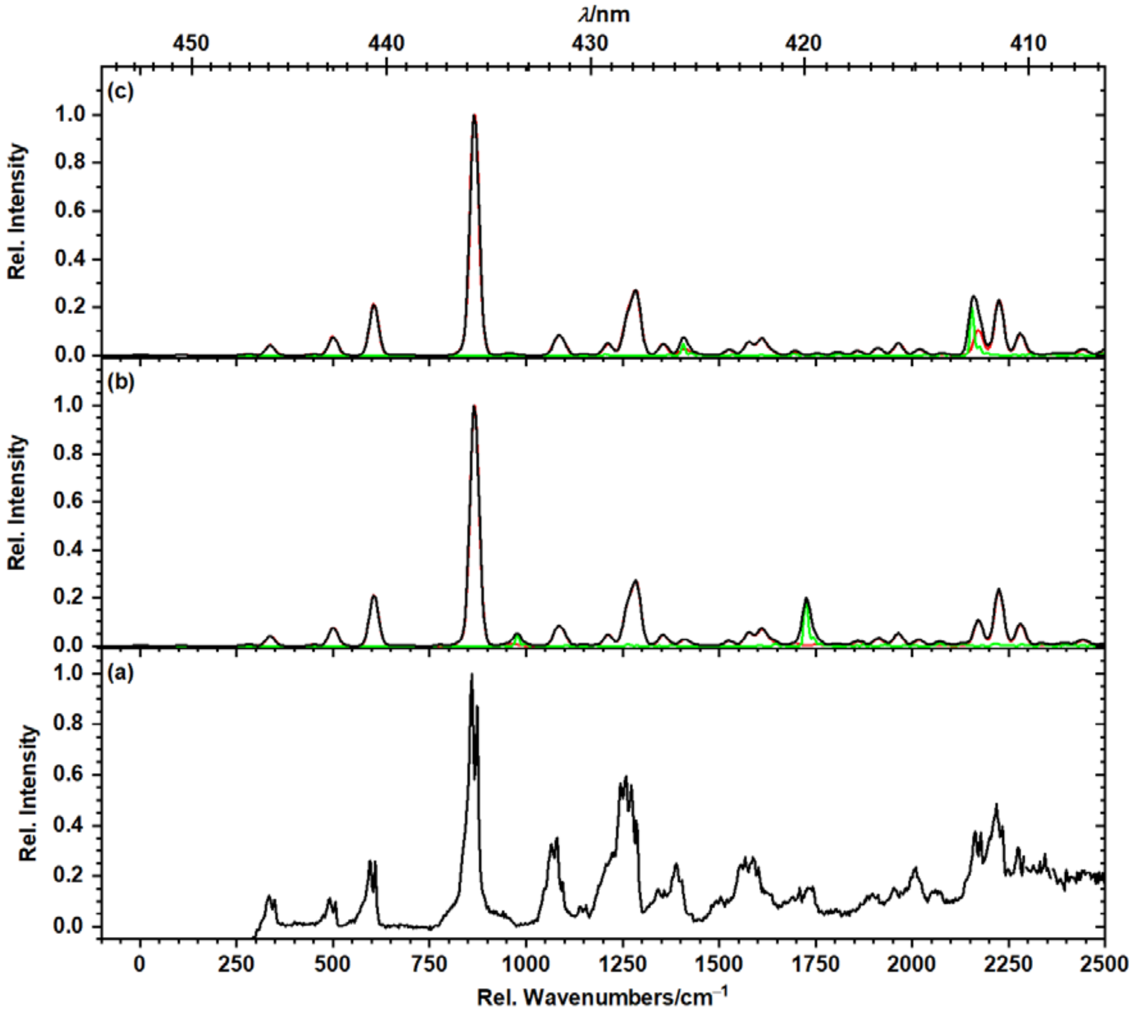

Figure S7. Comparison of experimental fluorescence excitation spectrum of *peri*-HBC isolated in solid *para*-H_2 with simulations. (a) Experimental spectrum. (b) Simulated $S_1 \leftarrow S_0$ (red) and $S_2 \leftarrow S_0$ (green) absorption spectra assuming the position of the 0_0^0 band of $S_2 \leftarrow S_0$ to be 775 cm^{-1} above the $S_1 \leftarrow S_0$ 0_0^0 band. (c) Assuming the position of the 0_0^0 band of $S_2 \leftarrow S_0$ to be 1205 cm^{-1} above the $S_1 \leftarrow S_0$ 0_0^0 band. The computed stick spectrum was convoluted with a Gaussian line shape of full-width-half-maximum (FWHM) 25 cm^{-1}. The black lines in panels (b) and (c) correspond to the sum of the simulated S_1 and S_2 absorption spectra; it overlaps mostly with the red line. Simulations were performed with the (TD-)B3PW91/6-311++G(2d,2p) calculations and wavenumbers were scaled by 0.982. The top abscissa is wavelength (nm) and the bottom abscissa is wavenumbers (cm^{-1}) above the origin of $S_1 \leftarrow S_0$ at 452.7 nm (22088 cm^{-1}).

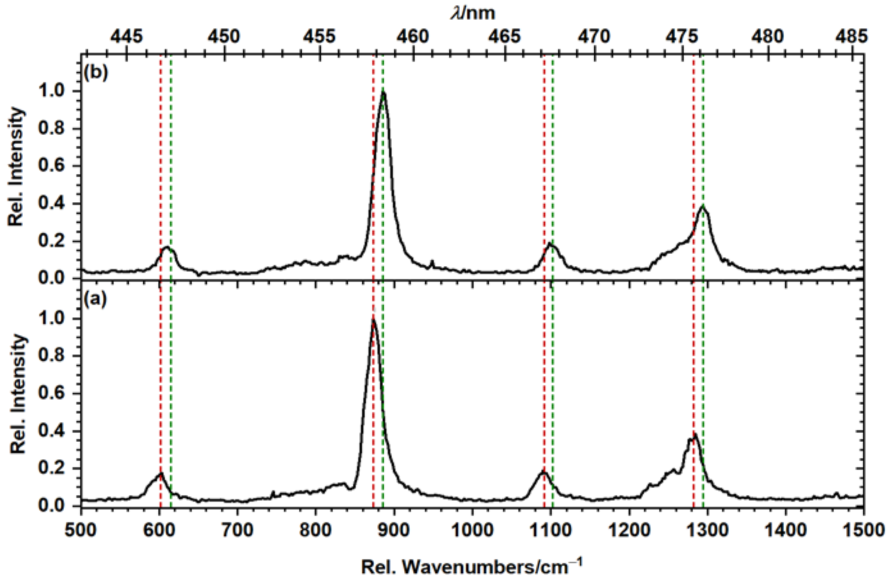

Figure S8. Partial dispersed fluorescence spectra of *peri*-HBC in solid *para*-H_2 after excitation at (a) 435.3 nm (22973 cm^{-1}) and (b) 435.5 nm (22962 cm^{-1}) corresponding to the two components of the most intense peak in the fluorescence excitation spectrum. Peak positions are marked in red (excitation at 435.3 nm) and green (excitation at 435.5 nm) illustrating the consistent shift of ~11 cm^{-1}. The bottom abscissa is wavenumbers (cm^{-1}) below the origin of $S_1 \rightarrow S_0$ at 452.7 nm (22088 cm^{-1}).
